# Interventions for Visual Field Loss After Acquired Brain Injury: A Systematic Review

**DOI:** 10.3390/life16081275

**Published:** 2026-07-31

**Authors:** Naiara Díaz-Marín, Carmen López-de-la-Fuente, Yolanda Marcén, Jorge Pérez-Rey, Carmen Bilbao, María José López-de-la-Fuente

**Affiliations:** 1 Department of Applied Physics, University of Zaragoza, 50009 Zaragoza, Spain; 679752@unizar.es (N.D.-M.); carmenbilbao@unizar.es (C.B.); 2 iHealthy Research Group, Aragon Health Research Institute (IIS Aragón), 50009 Zaragoza, Spain; 3 Department of Human Anatomy and Histology, University of Zaragoza, 50009 Zaragoza, Spain; yomarcen@unizar.es (Y.M.); jorge.perez@unizar.es (J.P.-R.); 4 PhysiUZerapy Health Sciences Research Group, Aragon Health Research Institute (IIS Aragón), 50009 Zaragoza, Spain; 5 Department of Ophthalmology, Hospital Universitario Miguel Servet, 50009 Zaragoza, Spain; 6 Department of Physiatry and Nursing, University of Zaragoza, 50009 Zaragoza, Spain

**Keywords:** acquired brain injury, visual field loss, visual rehabilitation, homonymous hemianopia, neuroplasticity, stroke, compensatory training, restorative therapy, substitutive therapy, neuromodulation

## Abstract

Acquired brain injury (ABI) frequently results in visual field loss, a disabling condition that negatively affects visual exploration, reading, mobility, activities of daily living, and quality of life. This systematic review aimed to synthesise current evidence on the effectiveness of rehabilitation interventions for visual field loss following ABI. The review was conducted according to PRISMA 2020 guidelines and prospectively registered in PROSPERO (registration number: CRD420261359820). Four electronic databases (PubMed, Scopus, Web of Science, and ProQuest) were searched for studies published between January 2015 and May 2026. Twenty-eight studies met the eligibility criteria, including randomised controlled trials, quasi-experimental studies, and case series. Interventions were classified as compensatory, substitutive, or restorative, the latter including neuromodulation techniques. Compensatory interventions provided the most consistent evidence of benefit, improving visual exploration, reading, mobility, activities of daily living, and vision-related quality of life. Restorative interventions produced more variable findings, with some studies reporting improvements in visual field sensitivity and stimulus detection, whereas evidence for substitutive approaches was limited because few eligible studies were identified. Overall, functional improvements were reported more consistently than objective visual field expansion. Current evidence supports visual rehabilitation as an important component of ABI management, although further high-quality research using standardised protocols and outcome measures is needed to establish the most effective interventions for different patient populations.

## 1. Introduction

Acquired brain injury (ABI) refers to brain damage occurring after birth and may result from stroke, traumatic brain injury, cerebral anoxia, encephalitis, or brain tumours [1,2]. ABI represents a major global public health burden. In 2021, stroke accounted for 11.9 million incident cases and remained a leading cause of death and disability worldwide [3]. Traumatic brain injury accounted for 27.16 million new cases in 2019 [4].

ABI can result in a broad range of motor, cognitive, communication, and sensory disturbances that negatively impact quality of life [5,6,7]. Among these, visual deficits are common after ABI and frequently overlooked. Around 60% of stroke survivors present with visual impairment, commonly involving reduced central vision and ocular motility abnormalities. Notably, visual field loss represents one of the most disabling consequences, alongside visual perceptual deficits [8]. In traumatic brain injury, approximately 69% of individuals display visual dysfunction involving accommodation, convergence, and ocular motility [5], while visual field loss affects around 18% of patients [9].

Despite their high prevalence, visual deficits are frequently underdiagnosed because visual screening is not routinely performed in many stroke services and symptoms may be subtle or difficult for patients to recognise [8,9,10]. Current recommendations emphasise the need for structured assessment and clear referral pathways to ensure appropriate management of visual impairment in individuals with ABI [11].

Among the various visual dysfunctions associated with ABI, visual field loss is clinically significant, functionally disabling, and more common in severe cases [5,8,9]. Homonymous hemianopia is the most common presentation, although quadrantanopia, scotomas, and more diffuse field defects may also occur. Visual field loss substantially compromises functional mobility, reading, visual exploration, visuospatial orientation, driving, employment, and other activities of daily living, reducing quality of life. Consequently, the management of visual field defects represents an important clinical challenge in neurorehabilitation after ABI [12,13].

Several interventions are available to address visual dysfunction after ABI and may be delivered individually or in combination depending on patient needs [5,14,15,16]. In individuals with visual field loss, rehabilitation approaches include compensatory scanning strategies, prism-based interventions, and restitutive therapies [17,18,19,20]. These approaches differ in their proposed mechanisms and therapeutic goals. Training-based approaches are partly supported by neuroplasticity, as repeated visual stimulation may improve visual function and promote cortical reorganisation.

Previous reviews have evaluated interventions for visual field defects after stroke; since then, several newer rehabilitation approaches, including software-based and virtual reality interventions, have emerged in recent years [21,22,23,24,25]. These technological advances have expanded the possibilities for visual rehabilitation by enabling more immersive, adaptive, and accessible training environments, together with more precise monitoring of patient performance. Consequently, digital rehabilitation has become an increasingly active area of research in recent years. Accordingly, an updated synthesis of the current evidence is warranted.

Despite their clinical relevance, available evidence regarding rehabilitation strategies for visual field loss after ABI remains heterogeneous due to substantial differences in interventions, outcome measures, and reported effects across studies. Therefore, this systematic review aimed to synthesise current evidence on interventions for visual field loss following ABI and their associated visual and functional outcomes to support evidence-based clinical decision-making.

## 2. Materials and Methods

This systematic review was conducted in accordance with the PRISMA 2020 statement. The study was registered in PROSPERO (registration number: CRD420261359820). A protocol was developed prior to data collection to ensure methodological transparency and consistency throughout the review process.

The following research question was formulated according to the PICO framework (Population, Intervention, Comparison, and Outcome): in individuals with visual field defects secondary to ABI (P), what is the effectiveness of compensatory, restorative, and substitutive visual rehabilitation interventions (I), compared with no intervention, standard care, sham treatment, or pre-intervention status when available (C), in improving visual field function, visual abilities, activities of daily living, and quality of life (O)?

### 2.1. Eligibility Criteria

Studies met the following inclusion criteria:

- Population: Patients with ABI of any aetiology presenting visual field loss;
- Study focus: Examined therapeutic approaches aimed at addressing visual field loss or associated visual abilities;
- Outcomes: Reported changes in the visual field, related visual abilities, functional performance, or quality of life;
- Study design: Randomised controlled trials, non-randomised studies, and observational studies including case series;
- Language: Articles published in English.
- Publication date: Studies published between 2015 and May 2026, inclusive.

Studies were excluded based on the following criteria:

- Studies focused exclusively on ocular pathologies unrelated to ABI;
- Case reports, systematic reviews, meta-analyses, expert opinions, epidemiological studies, and conference abstracts.

### 2.2. Search Strategy and Information Sources

A systematic search was conducted in PubMed, Scopus, Web of Science, and ProQuest. The final search was conducted on 7 May 2026. Search strategies were adapted to each database using controlled vocabulary and terms related to ABI, visual field loss, and visual rehabilitation, combined with Boolean operators and truncation symbols when appropriate.

The same search terms were used across all electronic databases to ensure comparability of the searches.

- (“Traumatic Brain Injury” OR “TBI” OR “Stroke” OR “Cerebrovascular Accident” OR “brain tumor”) AND (“Vision Therapy” OR “Visual Rehabilitation” OR “Vision Training” OR “Oculomotor Training” OR “Visual intervention” OR “substitution therapy” OR “prisms” OR “Visual restoration” OR “Visual scanning”) AND (“field loss” OR “hemianopia” OR “quadrantanopia” OR “Visual field defect” OR “homonymous hemianopia”) AND (“Intervention” OR “Neurorehabilitation”);
- (“Traumatic Brain Injury” OR “TBI” OR “Stroke” OR “Cerebrovascular Accident”) AND (“field loss” OR Visual field defect”) AND (“Intervention” OR “Neurorehabilitation”);
- (“Acquired brain injury” OR “ABI” OR “Traumatic Brain Injury” OR “TBI” OR “Stroke” OR “Cerebrovascular Accident”) AND (“field loss” OR “Visual field defect”) AND (“Restitution Therapy” OR “oculomotor training” OR “substitution therapy” OR “Visual restitution” OR “Visual scanning”);
- (“Acquired brain injury” OR “ABI” OR “Traumatic Brain Injury” OR “TBI” OR “Stroke” OR “Cerebrovascular Accident”) AND (“field loss” OR “Visual field defect”) AND (“Treatment” OR “Neurorehabilitation”).

In addition to the electronic search, supplementary searches were performed using citation searching, by screening the reference lists of included studies and identifying subsequent articles that had cited them.

### 2.3. Selection of Studies

**Figure 1:**
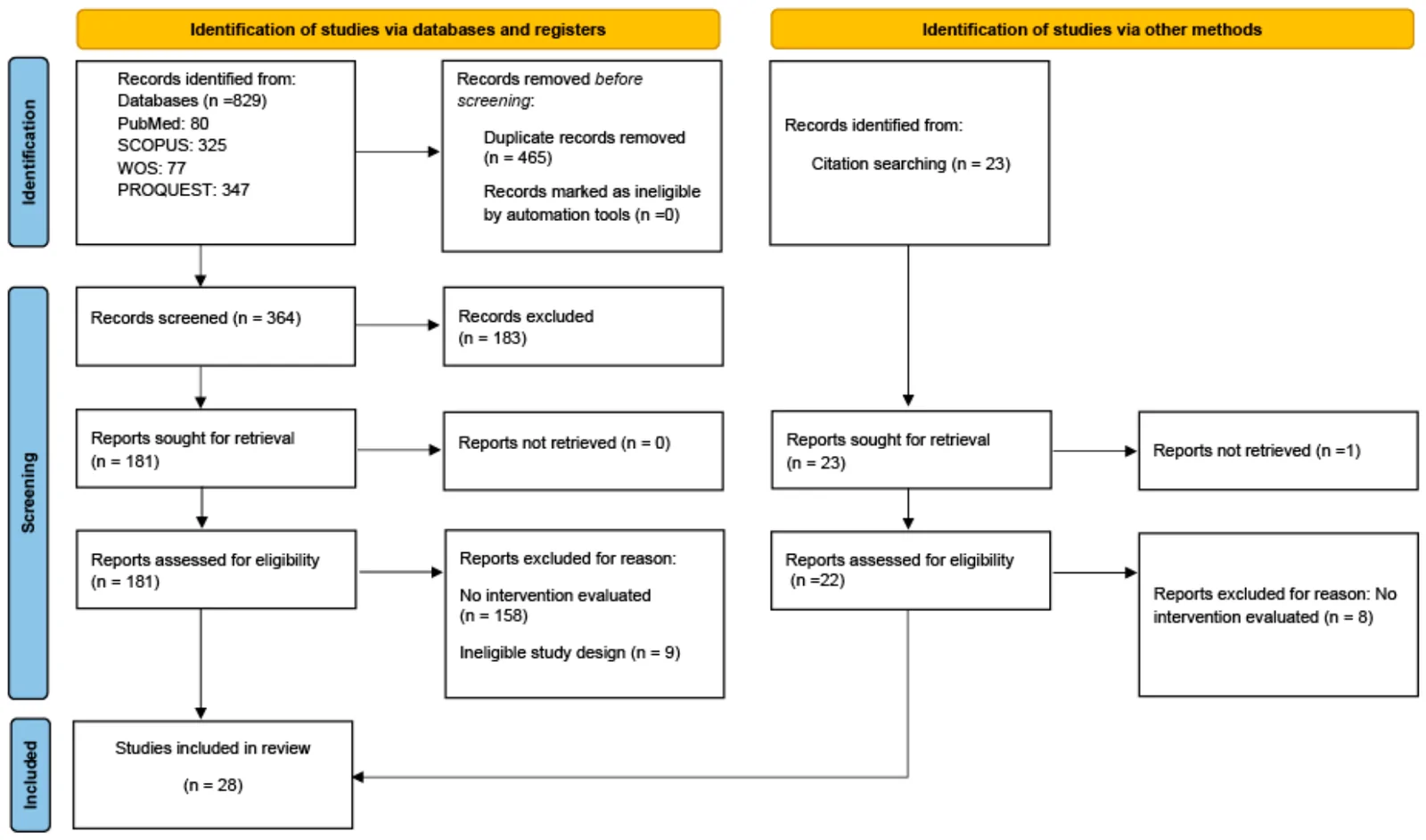
PRISMA 2020 flow diagram for new systematic reviews which included searches of databases, registers and other sources.

Three authors (ND, CLF, and MJLF) independently screened titles, abstracts, and full texts against the predefined eligibility criteria. The study selection process is summarised in the PRISMA flow diagram (Figure 1). Data were extracted by ND using a structured template and independently verified by CLF and MJLF, with any discrepancies resolved by consensus. For each eligible study, all data relevant to the predefined outcome domains were extracted. These included publication year, study design, sample size, population characteristics, study objectives, intervention type (where applicable), outcomes (visual field measures, related visual abilities, functional performance, and quality of life), and the main findings of each study. Only data explicitly reported in the original publications were extracted.

### 2.4. Quality Assessment

Methodological quality and risk of bias were independently assessed by two reviewers using critical appraisal tools appropriate to each study design. The PEDro scale was applied to RCTs [26], with scores reported descriptively without using score cut-offs [27]. The revised JBI Critical Appraisal Checklist was used for quasi-experimental studies [28], and the JBI Critical Appraisal Checklist for Case Series was applied to case series [29]. Consistent with JBI recommendations, methodological quality was interpreted narratively based on the main limitations and sources of bias. Any disagreements were resolved through discussion until consensus was reached.

### 2.5. Data Synthesis

Owing to the substantial clinical and methodological heterogeneity of the included studies, meta-analysis and formal assessment of reporting bias were not undertaken. Findings were synthesised narratively and grouped according to rehabilitation approach (compensatory, restitutive, and substitutive interventions). Due to the diversity of assessment tools and outcome measures, results were extracted as reported in the original studies, and the certainty of the evidence was interpreted narratively by considering the methodological quality and consistency of the included studies.

**Table 1:** Interventional studies on visual field rehabilitation after acquired brain injury.

Study (Author, Year)	Study Design	Sample/Population	Intervention	Outcomes/Measures	Assessment & Follow-Up Time Points	Main Results
De Haan et al., 2015 [17]	RCT (training vs. waiting-list control)	*N* = *49* VFD (HH/QD) >5 months post-injury (mostly stroke, TBI/other ABI); age 27–74 y. Exclusion: ocular disease affecting VF/VA, neglect, ataxia, sticky fixation, MMSE < 24, inability to walk 50 m, severe neuropsychological impairment	IH-CST 0816 scanning training 15. Supervised sessions (60–90 min). Over 10 weeks and structured homework. Delivered by OTs using standardised protocol.	Primary: Tracking Task; obstacle course; PPWS; DSR Secondary: Goldmann perimetry; FFS; dot counting; visual search; Radner reading; Questionaries (VFQ-25, IMQ, CVD)	Baseline and post-training (~13 weeks)	IH-CST improved peripheral detection in dual-task conditions, reduced obstacle contacts, and increased walking speed. Significant improvements were also reported in self-perceived mobility and vision-related difficulties. No changes in visual field or reading.
Elshout et al., 2016 [30]	RCT (Crossover study)	*N* = *27*; chronic stroke (>10 months) VFD (HH) 29–74 years	Two 8-week training rounds (1 h/day, 5 days/week); visual discrimination/visual restitution training (static or optical flow point stimuli); training applied to the affected hemifield and the intact hemifield.	Goldmann and Humphrey perimetry; ECSG Reading speed	Baseline, after first round, after second round	Defect-side training produced greater reductions in visual field defect (Goldmann) than intact-side training, with larger effects in those with greater baseline loss; Humphrey sensitivity improved in the trained hemifield, and reading. The speed increased significantly.
Grasso et al., 2016 [31]	Quasi-experimental	*N* = *10* Stroke (≈90%) chronic HH Mean age 49.8 y. Exclusion: neglect, hearing impairment	Multisensory audiovisual stimulation: 10 days, 4 h/day visual, auditory, and bimodal stimuli delivered mainly to the blind field.	Visual detection, Visual search (E-F test, Triangles test); reading speed; ADL inventory; oculomotor parameters; EEG (P3 amplitude)	Baseline, post-training and 8 months	Improvements in visual detection, visual search, reading speed, ADL scores, and oculomotor performance; EEG showed attentional reallocation toward the blind field; effects maintained at 8 months.
Sahraie et al., 2016 [32]	Quasi-experimental (pre-post; waiting-list control) EXPERIMENT 1	*N* = *32* Stroke VFD (HH); 10–64 weeks post-injury; mean age 56.6 y (17–82 y). Exclusion: visual neglect, depressive symptoms, moderate–severe attention, or memory impairment.	NeuroEyeCoach (NEC). Compensatory Visual Search Training (12 levels of pop-out, conjunction, and conjunction searches) is designed to enhance saccadic efficiency and develop a systematic search strategy for the VFD. Average of 13 sessions at home (approx. 45 min each) completed over 2 weeks. The control group received the intervention after their third assessment.	Objective: Time taken to complete a pen-and-paper cancellation task and reaction times (RT) on a computerised visual search task. Subjective: Questionnaire on Activities of Daily Living (ADL) (e.g., vision speed, bumping into obstacles).	A1: Baseline; A2: Post-8-week waiting period; A3: Post-2-week training; A4: 11-week follow-up	NEC training significantly enhanced visual search efficiency (27–31% faster RTs) following a stable 8-week baseline. Gains were maintained at the 11-week follow-up, supporting treatment- specific compensatory effects rather than spontaneous recovery.
	Quasi-experimental (pre-post) EXPERIMENT 2	*N* = *24* VFD (HH/QD) Time since onset ranged from 3 to 170 months Mean age 58.5 y	NeuroEyeCoach (NEC): compensatory visual search training programmed. 12 levels of increasing difficulty (pop-out, complex, and conjunction searches). Average of 13.1 sessions (clinical group; *n* = 9) or 14 sessions (online group; *n* = 15). Training was delivered via a self-adaptive automated system, with progression contingent on achieving an 85% accuracy threshold.	Inbuilt NEC assessments (Computer-based). Visual Search Task: Reaction Times (RT) and total number of errors; Cancellation Task: Median time of three measurements, clicking on targets. ADL self-report Questionnaire: 9-item scale (e.g., finding objects, crossing roads).	Baseline and post-training	Significant improvements in visual search efficiency were observed, with faster reaction times for both target-present (1125 ms to 957 ms) and target-absent trials, alongside a marked reduction in search errors (9.17 to 3.42). Self-reported ADL scores indicated a significant reduction in perceived visual disability from 18.47 to 13.21. In contrast, the computer-based cancellation task did not demonstrate sensitivity to change (*p* = 0.267).
Bergsma et al., 2017 [33]	Quasi-experimental (Within-subject trained vs. untrained ROI)	*N* = *24* (17 subacute Stroke;7 chronic) VFD range 18–75 years	Two 8-week training rounds targeting complementary ROIs (trained vs. untrained); discrimination-based visual restitution training, 1 h/day, 5 days/week.	Goldmann perimetry; ECSG Reading speed; GAS	Baseline, post-treatment, and long-term 14 months (subacute group)	Greater visual field recovery in trained ROI; training accounted for ~2/3 of total recovery; spillover ~1/3 of trained effect; reading speed and GAS improved proportionally to trained ECSG; effects remained stable at follow-up.
Cavanaugh & Huxlin 2017 [19]	Quasi-experimental (control group; blinded assessor)	*N* = *22* Chronic Stroke VFD (HH); mean age: 56–68. Exclusion: unreliable HVFs; ocular disease; neglect; neurological disease unrelated to occipital stroke; use of neuroactive drugs; inability to fixate precisely (>1° error); inability to complete psychophysical testing.	Restitutive visual perceptual learning (VPL) delivered within the blind field: motion discrimination, static orientation discrimination, or combined (“double”) training. 300 trials/day, 5 days/week, for 3–14 months (individualised duration). Training locations selected psychophysically within the defective hemifield. Trained G = 17 Untrained controls = 5	Primary: Humphrey perimetry (24-2 and 10-2): visual field area; PMD Secondary: fixation stability; Psychophysical discrimination thresholds; spatial distribution of VF changes; correlations with training dose and number of trained locations.	Baseline and post-training (3–14 months). Controls: Two HVFs 1.4–13.3 months apart.	Trained patients showed substantial visual field expansion (~108°^2^) compared with minimal change in controls (~16°^2^). PMD improved only in the trained group. Improvements correlated with training dose (sessions and trained locations), but not with age, lesion size, or time post-stroke. Gains extended beyond trained points into adjacent blind-field regions. Training type did not influence Outcomes after removal of one outlier.
Rowe et al., 2017 [34]	Pilot multicenter RCT (three-arm, single-blind)	*N* = *87* Stroke VFD (HH) Mean age 69 y	Prism glasses (Fresnel); ≥2 h/day, 6 weeks (*n* = 27) Visual search training; 30 min/day, 6 weeks (*n* = 25). Standard Care (control group, *n* = 22).	Primary: visual field (Goldmann/ Humphrey/Octopus) Secondary: VFQ-25; RMI; NEADL; EQ-5D/EQ-VAS; SF12; Radner reading Chart.	6, 12 and 26 weeks	No intervention improved visual field outcomes. Visual search training improves vision-related quality of life; prism therapy provides no benefit and causes frequent adverse effects. No differences were found in reading performance or other secondary outcomes.
Alber et al.; 2017 [35]	Open-label pilot study (non-randomised), case series with a matched retrospective control group	*N* = *14* HH post cerebral artery stroke.	Combined transcranial direct current stimulation (tDCS, 2 mA, 20 min per session) plus vision restoration training (VRT), administered over 10 daily sessions starting at ~66 ± 50 days post-stroke. 7 patients received the intervention (tDCS + VRT). 7 matched controls received standard rehabilitation	Visual field recovery measured by standard static perimetry (mean sensitivity threshold in decibels); percentage of visual field improvement; safety and tolerability (adverse effects, patient acceptance).	Baseline assessment before intervention and post-intervention evaluation after completion of 10 sessions; no long-term follow-up reported.	Both groups improved, but recovery was significantly greater in the tDCS/VRT group (36.73% ± 37.0%) compared with controls (10.74% ± 8.86%, *p* < 0.05). The intervention was safe and well tolerated, with only mild skin itching reported. Findings suggest potential superiority of combined tDCS + VRT over standard rehabilitation, supporting feasibility for larger randomised trials.
**Turton et al., 2018** [36]	Cohort study (pre-post; pilot feasibility study)	*N* = *9* stroke VFD (HH); 58–84 years Exclusion: severe cognitive impairment preventing participation or following instructions.	Compensatory visual scanning and search training delivered by occupational therapists; goal-oriented, ADL-related tasks practiced over 3 weeks (3 sessions/week).	Primary: Perception about ADL selected Secondary: VFQ-25; room-search task (head-mounted camera assessment).	Baseline and post-intervention (3 weeks)	Improvements were observed in ADL-related visual ability and room-search performance. VFQ-25 scores showed functional gains.
Crotty et al., 2018 [12]	RCT (single-blind)	*N* = *24* Stroke; 51 days post-injury. VFD (HH) Exclusion: aphasia, mobility < 35 m, MMSE < 25, VA < 6/18	Standardised NVT program: 3 weeks static scanning (9 sessions); 4 weeks mobility scanning (12 sessions) 3 sessions/week; 7 week	Primary: Mobility Assessment Course (MAC) Secondary: VSA, VSRT; VFQ-25; LVVFQ-48; MPAI; BIT.	Baseline, 7 weeks, and 3 months follow up	No differences were observed between groups in scanning performance while walking. The standardised program improved quality of life (VFQ-25 total and dependence scores). No significant effects were observed on MAC, reading, or VSA.
Smaakjær et al., 2018 [37]	Quasi-experimental, single arm pre–post study	*N* = *24* Stroke (HH + oculomotor dysfunction); median age 55.5 y.	Therapist-assisted vision therapy (10–12 weekly sessions + daily home training); targeting binocular vision, saccades, vergence, pursuit, peripheral awareness	Primary: COPM Secondary: Goffman tracing, reading speed, peripheral awareness, simple campimetry, MoCA, MFI-20, RBMT	Baseline and post-intervention (10–12 weeks)	Significant improvements in COPM, eye-movement control, reading speed, and peripheral awareness; increased functional safety. No formal perimetry field enlargement.
El Nahas et al., 2019 [38]	Open label pilot case series (pre-post)	*N* = *12* stroke VFD; ≥3 months post-injury 8 completed perimetry +1VFQ-25 Exclusion: lesions outside visual cortex; contraindications to rTMS; inability to complete perimetry	Navigated perilesional rTMS: 16 sessions (every other day), 10 Hz, 1000 pulses/session at 90% MT; 25 trains × 40 pulses, 20 s inter-train interval. Stimulation applied to four MRI-defined perilesional targets in bordering areas of residual vision.	Primary: Humphrey perimetry (MD, VFI) Secondary: VFQ-25; adverse events.	Baseline and post-intervention (after 16 rTMS sessions)	Significant improvement in Humphrey MD (−18.6 to −14.2 dB, *p* = 0.046); VFI increased non-significantly (45.6% to 58.2%). VFQ-25 scores improved significantly. MD gains ranged from +2.2 to +12.8 db. No correlation with chronicity or baseline severity. Minimal adverse effects.
Sahraie et al., 2020 [39]	Quasi-experimental	*N* = *296* HH, stroke 103, 101 left or right field loss 92 both fields	NEC: online 12 levels with 4 each of pop-out 3 episodes of 15 min per day 5 day per week 3 weeks (¾ 6 weeks) approx.	Living questionnaire. Practice session of 10 trials	Pre and post training	Age was a significant predictor of improved search errors, old patient’s larger improvements. Time between brain injury and intervention negatively influenced search reaction time. None of the factor could predict improved subjective reports of disability
Szalados et al., 2021 [40]	Quasi-experimental study conducted in a prospective cohort (within-subject control).	*N* = *426* (84% stroke) HH, neglect or both. Mean age 60 years.	Eye-Search web-based therapy: ramp–step eye-movement training 1200 trials (4 blocks × 400) completed at home.	Primary: Visual search reaction time (RT) into affected/unaffected field Secondary: PROM-ADL (“Finding things”); visual field test; neglect test.	Baseline, after 400, 800 and 1200 trials (average 20 days to T3)	Improved visual search RT (25–45%) except in neglect-only; parallel improvement in “Finding things”; small spontaneous visual field changes unrelated to visual search gains.
Cavanaugh et al., 2021 [41]	Randomised controlled trial (double-blind, sham-controlled).	*N* = *48* chronic post-stroke (HH). Age: 21–75 y.	Intervention group (*n* = 25): Computer-based visual retraining targeting the blind (hemianopia) visual field. Control group (*n* = 23): Identical computer-based training delivered to the intact visual field (sham intervention). Both groups same schedule.	Primary outcome: Visual field sensitivity assessed using Humphrey automated perimetry. Secondary outcomes: Visual discrimination performance and other vision-related functional measures.	Baseline (pre-intervention) and immediately after completion of the training program (8 weeks)	Significant within-group improvements in visual field sensitivity and task accuracy, with no significant between-group differences or improvements in quality of life, retinal thickness, or microperimetry.
Mena-García et al., 2021 [42]	Quasi-experimental (Control group; pre-post)	*N* = 40 Stroke (HH); time post-injury ~19 months. Mean age ~50 y. Exclusion: ocular disease, unrelated stroke, severe cognitive impairment, psychiatric or Neurological conditions affecting participation; uncorrected visual acuity deficits	3D Multitasking Compensatory Saccadic Training Program (3D-MCSTP): Twelve-week compensatory visual training combining saccadic, visuomotor, attentional and reading tasks using real three-dimensional objects. Four therapist-guided sessions, plus intensive home-based training (2 sessions/day, 5–7 days/week). Intervention *n* = 20; control *n* = 20;	Primary: Visual processing speed (Symbol Digit Modalities Test), visual attention and retention (Wechsler Memory Scale—Visual Reproduction), and reading speed. Secondary: Quality of life and functional impact (VFQ-25, SSQ-12), oculomotor performance, and Humphrey 30-2 visual field indices.	Baseline and post-intervention (12 weeks)	Significant improvements were observed in visual processing speed (−57%), visual attention and retention (+27%), reading speed (+10%), and quality-of-life measures. The comparison group showed no changes. Humphrey 30-2 indices improved slightly (+10%) but without significant differences between groups; Thus, no clinically meaningful visual field restoration was demonstrated.
Ajina et al., 2021 [20]	Quasi-experimental (Control group; pre-post)	*N* = *11* VFD (HH) stroke > 6 mo.	Neuro-Eye Therapy Program: daily home-based training (approx. 25 min) for 3–6 months. A temporal 2-AFC detection of drifting Gabor patches in the blind field, adjusted to maintain difficulty. Training group: *n* = 7 (mean age 61.3 y). Control group: *n* = 4 (mean age 49.5 y).	Psychophysical outcomes: contrast sensitivity (Gabor detection), motion detection (speed), and motion direction discrimination (coherence/speed). Humphrey perimetry (30-2 full threshold); fMRI (BOLD signal in area V5/hMT) and voxel-based morphometry (grey matter volume).	Baseline and post-training (3 to 6 months); controls assessed at comparable intervals, but not for all outcome measures.	Psychophysical visual sensitivity improved significantly in the trained group, whereas controls showed no comparable changes. Training was associated with focal gains in Humphrey perimetry within the trained blind-field region, alongside functional enhancements in occipital area V5/hMT and structural increases specifically in hippocampal grey matter volume, supporting compensatory neuroplasticity rather than spontaneous recovery.
Metzler et al., 2021 [43]	Retrospective chart audit (case series)	*N* = *49* post-stroke >13 mo. (VFL) >6 mo. (perceptual impairments)	Individualised compensatory visual training (visual scanning, functional tasks, computer-based exercises and home practice). (29 with VFL; 20 with visual-perceptual impairments).	Canadian Occupational Performance Measure (COPM); Useful Field of View (UFOV); Dynavision Performance Assessment Battery; demographic and treatment characteristics extracted from clinical records.	Baseline and discharge (mean ≈ 13–14 treatment sessions).	Visual retraining was feasible in routine. Improvements were observed in all outcome measures. Significant improvements with large effect sizes were found for several Dynavision measures in patients with VFL and for UFOV divided attention in patients with visual-perceptual impairment. Compensatory visual rehabilitation may improve functional visual performance. Findings should be interpreted cautiously because of the retrospective, uncontrolled study design.
Leitner et al., 2023 [23]	Quasi-experimental (pre-post)	*N* = *16* stroke V1/optic radiations lesions; VFD (HH) Mean age 55 y. Exclusion: neglect, cognitive deficits	Restitution training: VR goggles (SVFT); 5 months, 2 × 30 min/day, 6 days/week.	Primary: Eye tracking-based Visual field Analysis (EFA); secondary: VAS subjective visual field rating	Intermediate ~2 mo.; pre-post (mean 154 days)	No objective perimetric improvement (EFA); subjective improvement of the VAS (+11.5%); Findings interpreted as a placebo effect; no evidence of Restitution Training-induced neuroplasticity.
Gupta et al., 2024 [44]	Quasi-randomised clinical trial (alternate allocation)	*N* = *50* VFD (HH) chronic >6 mo. (stroke, TBI and tumour) Mean age 46 y. Exclusion: ocular pathologies other than cataracts; multiple disabilities; cognitive dysfunction.	Pre-phase: All received yoked prisms (*n* = 50) for 1 month. Group 1 (*n* = 15): Yoked prisms. Group 2 (*n* = 15): Yoked prisms + 10 sessions (1 h each) of in-office. Visual search training with SVI.	Visual search performance (SVI: reaction time, completion time, accuracy), reading speed (Reanalyze), DEM ratio, and visual memory.	Baseline and immediate post-intervention	Combined therapy (yoked prisms + SVI) yielded significantly greater visual search improvements than prisms alone. Enhancements in reading speed and oculomotor performance were also observed. These gains are consistent with compensatory mechanisms that improve scanning efficiency and attention.
Namgung et al., 2024 [24]	RCT (Multicentre double-blind)	*N* = *75* chronic stroke VFD (>6 mo.) (HH/QD) Mean age 52 y. Exclusion: unreliable VF results, cognitive, or other ophthalmic diseases.	Nunap Vision (NV): VR-based visual perceptual learning (VPL) targeting defective field. Comparator: NV-Control (NV-C), VPL applied to central field. 60 sessions: 348 trials/day; 5 days/week, 12 weeks.	Primary: HVF improved visual area ≥6 dB (defective and whole field) Secondary: Mean total deviation (MTD) scores; VPL task performance	Baseline and 12 weeks (post-training)	Both groups achieved clinically significant improvements in the visual area, with no differences between groups; MTD improved only within the NV; both groups showed improvements in VPL performance; high compliance and a good safety profile.
Diana et al., 2025 [45]	RCT (three-arm, double-blind, sham-controlled)	*N* = *18* VFD (HH) chronic (stroke, TBI, AVM) Mean Age 54 y. Exclusion: neglect, severe cognitive or attentional deficits, epilepsy, implanted devices, psychiatric disorders, visual acuity < 20/40, ocular diseases, pregnancy	AVT multisensory compensatory training: 10 days (2 h/day). Concurrent tDCS (occipital, parietal, sham-placebo) All groups received identical AVT	Primary: Visual detection (unimodal/multimodal) in blind hemifield; visual search (E-F test, Triangles test). Secondary: ADL questionnaire (compensatory visual behaviours); eye- movement measures	Baseline, post-training, 1 month, and 4 months.	Audiovisual compensatory training improved visual search performance and activities of daily living across all groups. Adjunctive tDCS accelerated training-related improvements, while occipital tDCS produced greater and sustained improvements in visual detection within the blind hemifield for up to 4 months compared with sham stimulation.
Namgung et al., 2025 [25]	RCT Multicentre	*N* = *82* chronic stroke (≥3 mo) VFD (HH/QD) Median age 52 y.	Personalised VR-Based VPL (orientation/rotation discrimination with Gabor stimuli). 360 trials/day, 5 days/week, 12 weeks.	Primary: Humphrey Visual field (HVF 30-2) Secondary: PMD (whole field); MTD (defective hemifield)	Baseline and 12 weeks	The training resulted in significantly greater improvements in the full-field and defective-field visual areas (≥6 dB) than in the control group; PMD and MTD improved only in the training group. High adherence and a good safety profile.
Varalta et al., 2025 [46]	Cohort study (home-based vs. clinic-based NEC training)	*N* = *126* VFD (HH) after ABI (mostly stroke). Exclusion: incomplete NEC training, inability to complete assessments	NeuroEyeCoach (NEC) visual search training: adaptive compensatory program; domiciliary unsupervised training vs. clinic-based supervised training. Standardised NEC protocol completed by all participants. Home group *n* = 95 (~58 y); Clinic group *n* = 31 (~60 y)	Primary: Visual search reaction time (RT); visual search errors. Secondary: Cancellation task RT; subjective disability questionnaire	Pre-post training	Both groups showed significant improvements in visual search RT, errors, and cancellation performance. Objective gains were larger in the clinic-based group, while subjective disability improved similarly in both groups.
Rowe et al., 2025 [47]	Multicentre RCT (two-arm, double-blind, sham-controlled)	*N* = *161* VFD (HH) 4–26 weeks post-stroke Mean age 67 y. Exclusion: neglect; severe cognitive/language impairment; ocular disease affecting VF; unstable medical condition; inability to comply with training.	Visual Scanning Training (VST): compensatory ≥ 30 min/day, 7 days/week for 6 weeks (minimum 20 h) Compared with sham training (slow eye-tracking tasks) Both self-administered at home with weekly remote support.	Primary: VFQ-25 Secondary: NEADL; EQ-5D-5L/EQ-VAS; BIVI-IQ, Visual scanning (RT and accuracy); Esterman visual Field.	Baseline, 6–12–26 weeks	Both groups showed significant improvements in VFQ-25, NEADL, EQ-5D, BIVI-IQ, visual scanning performance, and Esterman VF, with no differences between VST and sham. Improvements were similar across all outcomes, indicating no superiority of VST over sham (Sham includes active oculomotor engagement and frequent support).
Toh et al., 2026 [48]	Single-arm, non-randomised clinical trial	Drivers with HH. *N* = *16* > 3 months post-injury Median age = 52.5	Scanning training 3 sessions 7 1 h. Reinforcement-based head-scanning in driving simulator	Primary: proactive blind-side scanning and blind-side hazard detection. Pretraining, 1-week post-training, and 1-month post-training.	Pre-post training	Sessions improved blind-side head scanning and hazard detection in drivers. Benefits for at least one month. Need further evaluation in a randomised controlled trial.
Otero-Currás et al., 2026 [49]	Controlled parallel-group design	*N* = *52* ABI, Median age = 51	Dichoptic vision therapy, based on virtual reality systems. 6 sessions 27 training group, 25 control group	Primary: perimetric visual field indices and oculomotor reaction times. Secondary: Brain injury vision symptom survey	Pre-post training	No significant improvement in perimetric visual field indices. Experimental group reported a reduction in visual symptoms.
Jefferson et al., 2026 [50]	Pilot study	*N* = *10* Media age = 65. Stroke with HH	Visuo-cognitive training (TVT) using Senaptec sensory station	Visual cognition, patient-reported outcomes, usability and acceptability (pre and post intervention) Secondary: qualitative interviews.	Pre-post training	TVT demonstrated with selective improvements in visual search function and vision-related quality of life.

In alphabetical order: ADL: Activities of Daily Living, AVM: Arteriovenous Malformation, AVT: Audiovisual training (multisensory compensatory visual training), BIT: Behavioural Inattention Test, BIVI-IQ: Bangor Irregular Visual Information-Impact Questionnaire, COPM: Canadian Occupational Performance Measure, CVD: Cerebral Visual Disorders questionnaire, DSR: Dual-to-Single Task Ratio, DEM: Development eye movement, ECSG: Equivalent Cortical Surface Gain, COPM: Canadian Ocupational performance measure, EQ-5D/EQ-VAS: EuroQol 5 dimensions questionnaire/EuroQol Visual Analogue Scale, FFS: Functional Field Score, GAS: Goal Attainment Scaling, HH: Homonymous Hemianopia, IH-CST: In-Home Compensatory Scanning Training, IMQ: Independent Mobility Questionnaire, LVVFQ-48: Low-Vision Visual Functioning Questionnaire–48 items, MTD: Mean total Deviation, MPAI: Mayo–Portland Adaptability Inventory. Assesses functional abilities, psychosocial adjustment, and participation after brain injury, NEADL: Nottingham Extended Activities of Daily Living Scale, OT: Occupational Therapy, PMD: Pattern Mean Deviation, PPWS: Percentage Preferred Walking Speed, PROM: patient-reported outcome measure, QD: Quadrantanopia, RMI: Rivermead Mobility Index, SF-12 Shot Form-12 Health Survey, SVFT: Salzburg Visual Field Trainer, tDCS: transcranial direct current stimulation, TVT: Visuo-cognitive training, FOV: Useful Field of View test, UFOV: Useful field of view, VAS: Visual Analog Scale, VFD: Visual Field Defect, VFQ-25: Visual Function Questionnaire-25, VPL: visual perceptual training, VR: Virtual reality, VSA: Visual Scanning Assessment, VSRT: Pepper Visual Skills for Reading Test, WMS-R: Wechsler Memory Scale-Revised.

## 3. Results

### 3.1. Study Selection

A total of 829 records were identified through database searching (PubMed, *n* = 80; Scopus, *n* = 325; Web of Science, *n* = 77; and ProQuest, *n* = 347). After removing 465 duplicates, 364 records were screened by title and abstract; 183 were excluded. Full-text assessment was performed for 181 reports identified through database searching, and for 22 reports identified through citation searching. During the eligibility assessment, 158 database reports and 8 reports identified through other sources were excluded because they did not evaluate rehabilitation interventions relevant to the review question, while a further 9 reports were excluded because their study design did not meet the predefined inclusion criteria. Ultimately, 28 studies met the eligibility criteria and were included in the qualitative synthesis (Figure 1).

### 3.2. Study Characteristics

The 28 included studies comprised nine randomised controlled trials [12,17,24,25,30,34,41,45,47], eighteen quasi-experimental studies, including pre–post, pilot, and non-randomised controlled studies [19,20,23,31,32,33,35,36,37,38,39,40,42,44,46,48,49,50], and one retrospective case series [43]. Sahraie et al. [32] reported two independent experiments; these were analyzed separately in this review. Table 1 summarises the key characteristics of the included studies.

The included studies showed substantial heterogeneity in study design, participant characteristics, intervention protocols, and outcome measures. Across all studies, the cumulative sample comprised approximately 1905 participants, with a mean sample size of 68.0 (range: 9–426). The mean participant age was 56.9 years (range: 10–82 years). Intervention duration varied considerably, ranging from intensive short-term protocols lasting several days to programmes extending over several months, with treatment periods ranging from 3 days to 14 months. Session duration was also highly variable, although most interventions involved sessions lasting between 30 and 60 min.

Most participants had chronic visual field defects following ABI, predominantly homonymous hemianopia secondary to stroke, although quadrantanopia and broader visual field defects were also represented. Most studies focused exclusively on stroke-related visual field loss. Only five studies included participants with ABI of mixed aetiologies, including stroke, traumatic brain injury, and brain tumours [17,44,45,46,49]. Some studies also included subacute patients (1–6 months after ABI) [20,23,32,39,44,45,50], and six articles included mixed chronic and subacute populations [12,17,24,25,33,34]. Considerable heterogeneity was observed regarding lesion aetiology, chronicity, severity of visual impairment, and functional disability.

The reviewed interventions comprised compensatory, restitutive, and substitutive rehabilitation strategies. Compensatory approaches were the most frequently investigated (reported in 16 studies, comprising 17 intervention cohorts) [12,17,31,32,36,37,39,40,42,43,45,46,47,48,49,50], followed by restitutive interventions (*n* = 7) [19,23,24,25,33,38,41]. No study investigated a substitutive approach in isolation. Three studies evaluated both compensatory and restitutive interventions [20,30,35], whereas two others combined compensatory rehabilitation with substitutive strategies, including prism-based approaches [34,44].

Three of the aforementioned studies incorporated neuromodulation techniques as part of the intervention, including repetitive transcranial magnetic stimulation (rTMS) [38] and transcranial direct current stimulation (tDCS) [35,45]. Neuromodulation was applied as an adjunct to compensatory audiovisual training in one study [45] and to restitutive rehabilitation interventions in two studies [35,38].

A wide variety of visual stimuli and training modalities were employed, including computer-based visual search tasks, reading exercises, eye movement training, virtual reality environments, dichoptic stimulation, driving simulation tasks, audiovisual feedback, prism adaptation, and repetitive rTMS. Outcome measures varied substantially across studies and included objective visual field assessments, visual scanning performance, reading ability, oculomotor function, activities of daily living, visual attention, driving-related performance, and vision-related quality of life measures. Improvements in quality-of-life measures were reported in 12 of the 28 studies [12,17,23,32,34,36,38,39,42,47,49,50].

Sixteen of the seventeen studies evaluating only compensatory rehabilitation reported improvements in visual performance, scanning strategies, reading ability, and functional visual outcomes [17,20,31,32,36,37,39,40,42,43,44,45,46,47,48,50]. Similarly, one of the two studies evaluating compensatory and substitutive combined treatment also reported significant gains [44]. Only Rowe et al. [34] and Crotty et al. [12] failed to demonstrate significant functional improvement following treatment. Visual field outcomes were reported in eight compensatory studies [17,34,37,40,42,47,49,50]. Overall, no clinically meaningful improvements in visual field measures were observed following compensatory interventions. The only exception was Mena-García et al. [42], who reported small improvements of limited clinical relevance.

Only two studies investigating prism-based interventions met the inclusion criteria. In both cases, prism therapy was not evaluated as a stand-alone intervention but rather compared with compensatory training or combined with compensatory rehabilitation approaches [34,44].

Eight of the ten studies assessing restitutive rehabilitation reported significant within-group improvements in perimetric sensitivity (dB) [19,20,24,25,30,35,38,41], while six also quantified objective visual field expansion using square degrees (deg^2^) or equivalent cortical surface gain (ECSG) [19,24,25,30,33,35]. Three studies showed greater objective improvements than their respective control conditions [19,25,30], whereas two studies [24,41] did not find significant between-group differences following intervention. Leitner et al. [23] found no objective perimetric changes. Several studies also described gains in visual and functional abilities.

To facilitate comparison across studies, a table summarising the main outcomes of each included study is provided in Appendix A (Table A1).

### 3.3. Quality of Evidence and Risk of Bias

Most RCTs demonstrated good methodological quality, with only one study scoring below 6 on the PEDro scale (Table 2). The PEDro items most frequently not fulfilled were therapist blinding [12,17,24,30,34,41,45,46,47], participant blinding [25,30,41], and intention-to-treat analysis [17,25,30,34,41,47].

Risk of bias assessment varied across studies (Table 3). Only eight studies included an independent control group [19,20,32,35,42,44,46,49]. To mitigate the absence of an independent control group, several studies used within-subject comparison strategies, including comparisons between trained and untrained regions of interest [33] and between the affected and intact visual field [31,39,40,48].

The chronic stage of most participants, along with brief interventions, reduced the risk that the observed improvements were attributable to spontaneous recovery or external co-interventions [19,20,31,32,36,37,38,39,40,48,49,50].

Four studies incorporated repeated assessments both before and after the intervention to support baseline stability and evaluate treatment effects [31,32,33,44]. Five studies included repeated assessments at only one phase of the study, either before [19] or after the intervention [23,40,42,48]. Most studies employed objective, standardised, or automated outcome measures, thereby minimising measurement bias.

The included case series [43] achieved a high score on the JBI case series appraisal tool (Table 4) and provided valuable ecological validity by reflecting routine outpatient clinical practice. However, the retrospective design, non-standardised outcome assessment across participants, and substantial missing data for some functional outcomes increased the risk of bias.

Overall, the included studies showed the most consistent findings for compensatory interventions. Findings for restorative interventions were more heterogeneous, whereas evidence for substitutive interventions and adjunctive neuromodulation was limited.

## 4. Discussion

This systematic review synthesised current evidence on rehabilitation strategies for visual field loss following ABI, including compensatory, substitutive, and restorative interventions. Considerable heterogeneity was observed across studies regarding intervention protocols, patient characteristics, treatment duration, and outcome measures. Consequently, methodological heterogeneity complicates direct comparisons between studies and limits conclusions regarding treatment effectiveness; however, the available evidence may still inform clinical decision-making and visual rehabilitation strategies according to patient needs.

The methodological quality of the included RCTs was generally good, with most studies scoring ≥7 on the PEDro scale. Blinding of therapists and participants was the criterion least frequently fulfilled, reflecting the practical constraints of non-pharmacological rehabilitation trials. In contrast, the quasi-experimental studies exhibited greater methodological heterogeneity. The absence of an independent control group was the predominant source of bias, although several studies partially addressed this by employing within-subject comparison strategies. The use of objective, standardised, and automated outcome measures helped minimise measurement bias.

The interpretation of the available evidence should also consider several important limitations. Although the included studies provide encouraging findings, relatively few high-quality randomised controlled trials have been conducted, and many studies involved small sample sizes. These limitations reduce statistical power and restrict the generalisability of the findings. Furthermore, the considerable variability in participant characteristics, intervention protocols, treatment dosage, and outcome measures makes direct comparison between studies difficult and limits the strength of the conclusions that can be drawn.

### 4.1. Interventions

#### 4.1.1. Compensatory Treatment

Compensatory therapies for visual field loss primarily aim to improve functional adaptation by enhancing visual search, scanning patterns, and environmental awareness rather than restoring the damaged visual field. Findings from the included studies suggest that systematic visual search training and structured scanning programs improve visual exploration efficiency, mobility, reading performance, and activities of daily living [12,13,36,43,49,51]. Adjunctive tDCS was associated with faster training-related improvements and enhanced visual detection in the blind hemifield [45].

Eye movement and visual scanning-based interventions were consistently associated with improvements in visual exploration tasks and functional performance, whereas gains in reading appeared less consistent across studies [12,17,31,36,42]. Importantly, these functional gains are often observed despite limited perimetric changes, suggesting that the treatment effects may reflect adaptive compensatory oculomotor strategies [52,53].

The clinical relevance of these adaptive mechanisms is further supported by studies evaluating complex real-world activities. Howard et al. [54] observed that individuals with homonymous hemianopia who met driving reassessment criteria demonstrated more efficient compensatory scanning behaviours. Similarly, Toh et al. [48] reported that structured head-scanning training combined with real-time auditory feedback improved hazard detection and reaction times during driving tasks. More recently, telerehabilitation and digital interventions facilitating access to compensatory training have demonstrated promising results in both paediatric and adult populations [49,50,55].

These findings highlight the value of functional outcomes and the potential of more accessible visual rehabilitation approaches.

Although objective visual field recovery remains an important therapeutic goal, one of the most clinically relevant findings of this review is that improvements in functional outcomes were reported far more consistently than changes in perimetric measures. Across the included studies, rehabilitation frequently enhanced reading performance, visual exploration, mobility, activities of daily living, and vision-related quality of life, even in the absence of measurable visual field expansion [12,17,31,36,42]. These findings reinforce the importance of evaluating rehabilitation success using functional and patient-centred outcome measures rather than relying solely on objective perimetric assessments.

#### 4.1.2. Substitutive Treatment

Substitutive therapies for visual field loss following ABI aim to redirect visual information from the affected hemifield toward preserved visual areas using optical devices, thereby facilitating environmental awareness without directly restoring the underlying neural damage. An important finding of the present review is the limited amount of evidence available for this treatment category, as only two studies met the inclusion criteria and both evaluated prism use in conjunction with, or in comparison with, compensatory interventions rather than as an isolated treatment strategy [34,44].

The available findings suggest that prism therapy alone may provide modest benefits, mainly related to visual orientation and awareness. However, its effectiveness appears to improve when combined with active visual training. Rowe et al. [34] reported no significant differences in objective visual field outcomes. They observed greater improvements in vision-related quality of life among patients receiving visual search training compared with prism treatment. In addition, prism use was also associated with a high frequency of adverse events, particularly headaches. A similar pattern was observed by Gupta et al. [44], who found that participants receiving combined treatment with yoked prisms and structured visual search training demonstrated better visual search performance than those receiving prisms alone, with improvements in reaction time, reading performance, and oculomotor measures. Controlled clinical trials have evaluated their efficacy in comparison with standard occupational therapy and visual search training, reporting modest but functionally meaningful improvements in spatial orientation and mobility [51,52].

These findings suggest that the potential contribution of substitutive therapies may rely less on optical field displacement itself and more on their integration with active rehabilitation strategies that promote adaptive visual behaviours.

#### 4.1.3. Restorative Treatment

Restorative interventions are designed to promote partial visual field recovery through stimulation of residual visual pathways and facilitation of neuroplastic changes [20,23,32]. Unlike compensatory approaches, these interventions are designed to induce objective improvements in visual field sensitivity and detection. It should be noted that evidence from prospective studies suggests that partial spontaneous recovery of visual fields occurs predominantly during the early period after acquired brain injury [56,57].

Within restorative rehabilitation approaches, two main therapeutic strategies were identified. One group of interventions is based on repetitive visual stimulation and perceptual training, in which patients are required to maintain central fixation while responding to stimuli presented in specific regions of the impaired visual field [19,23,24,25,30,32,33,41]. A second group of restorative interventions involves neuromodulation techniques designed to enhance cortical excitability and neuroplasticity. These include rTMS and tDCS [35,38].

Compared with compensatory approaches, restorative interventions based on repetitive visual stimulation and perceptual training showed greater variability in both treatment protocols and reported outcomes. Differences in stimulus characteristics and target training regions, ranging from border-zone stimulation to residual functional areas, were observed [19,23,24,25,33,38]. Although several studies reported improvements in visual sensitivity, stimulus detection, and local visual field measures, the magnitude and consistency of these effects varied considerably across studies [24,25,30,33].

Recent technological developments, including digital environments and virtual reality, have enabled greater precision in stimulus presentation, improved patient engagement, eye-tracking-based gaze monitoring, and more accurate performance assessment. Preliminary findings suggest promising results regarding improvements in peripheral detection and visual response speed [24,25].

Evidence regarding neuromodulation remains limited. El Nahas et al. [38] reported that navigated perilesional rTMS improved visual field sensitivity and patient-reported visual function after 16 sessions, although with marked interindividual variability. Alber et al. [35] combined computer-based vision restoration training with adjunctive anodal tDCS. Compared with standard compensatory rehabilitation, combined vision restoration training and tDCS produced greater improvements in perimetric sensitivity and a greater reduction in absolute visual field defects. Overall, small samples, heterogeneity, and limited controlled evidence preclude firm conclusions on the efficacy of neuromodulation in visual field rehabilitation.

Current evidence suggests restorative treatment responses may depend substantially on individual biological characteristics rather than solely on the intervention itself. Residual neural activity, attentional network integrity, lesion characteristics, and time since injury have been associated with differences in rehabilitation outcomes [20,58,59]. These factors may partly explain the considerable heterogeneity in individual responses observed across studies and reinforce the importance of personalised treatment approaches. Further research is needed to determine which patients are most likely to benefit from restitutive therapies. It is also essential to clarify whether and how specific brain regions can be targeted depending on the location and extent of the lesion, as well as to develop standardised and clearly defined clinical protocols.

An additional finding across restorative studies is the frequent discrepancy between objective visual field measurements and reported subjective functional outcomes [20,23,38,48]. This suggests that improvements associated with restorative approaches may not necessarily reflect complete recovery of visual field function but could partly arise from adaptive behavioural mechanisms or more efficient use of residual visual processing.

Neuroimaging studies provide further insight into the mechanisms underlying variability in restorative treatment response. Halbertsma et al. [59], in a follow-up analysis of the cohort described by Elshout et al. [30], reported that stronger functional connectivity between the anterior precuneus and occipital visual networks was associated with greater responsiveness to visual restitution training. Ajina et al. [20] found that greater baseline activity in area V5/hMT was associated with larger improvements in visual sensitivity.

Additionally, Halbertsma et al. [58] reported that micro-probing was more sensitive than standard perimetry for detecting preserved activity within clinically blind regions. The authors emphasise that identifying spared visual pathways may help optimise rehabilitation and guide training approaches. However, the high cost and limited accessibility of advanced neuroimaging techniques may limit their use in routine clinical practice [20,23,38,48].

Among the most consistent findings, compensatory interventions demonstrated the most reproducible functional benefits, whereas restorative approaches showed more variable outcomes. Functional improvements, particularly in visual exploration, reading performance, mobility, activities of daily living, and quality of life, were more consistently reported than objective changes in visual field measurements.

The predominance of stroke populations within the included studies should also be considered when interpreting the findings of this review. Only five studies included participants with ABI of mixed aetiologies [17,40,44,45,46]. Four of these investigated compensatory interventions [17,40,45,46], while one study [44] combined compensatory and substitutive (prism-based) approaches. Consequently, current evidence remains largely derived from stroke-specific populations, limiting the ability to extrapolate findings to other forms of ABI.

This imbalance in the available evidence should be acknowledged when interpreting the findings of this review. Although stroke is the leading cause of acquired visual field loss, evidence regarding rehabilitation following traumatic brain injury, brain tumours, and other causes of acquired brain injury remains scarce. Consequently, the effectiveness of current rehabilitation approaches in these populations is still uncertain, highlighting the need for future studies specifically targeting these underrepresented groups.

Overall, post-stroke visual field loss is a complex, multifactorial condition whose early identification and comprehensive characterisation are critical for accurate prognosis and effective rehabilitation planning. Across the studies included in this review, improvements following visual rehabilitation were not limited to visual field measures alone; they frequently extended to related functions, including reading, visual exploration, spatial orientation, or daily functioning. Optimised scanning and search mechanisms, as well as targeted perceptual stimulation, translate into better performance in daily tasks, greater autonomy, and reduced risk of domestic and mobility-related accidents [25,36,53].

Moreover, these functional improvements are closely associated with improved quality of life. They reduce dependency, increase patients’ confidence in navigating their environment, and enhance social participation. Evidence suggests that even small improvements in visual efficiency or spatial perception can lead to clinically meaningful benefits. This highlights the importance of comprehensive assessment and individualised interventions targeting both sensory recovery and functional adaptation [8,22].

This review has several limitations. Despite a comprehensive search across major databases and manual reference screening, only English-language studies were included, potentially excluding relevant evidence. Second, owing to substantial heterogeneity in study designs, intervention protocols, participant characteristics, and outcome measures, a meta-analysis was not feasible. Third, the available evidence was dominated by stroke populations, limiting the generalisability of the findings to other ABI aetiologies. In addition, many studies included small sample sizes, limiting the robustness of the available evidence. Finally, few studies evaluated substitution therapies and neuromodulation; conclusions regarding these interventions should be interpreted with caution. Therefore, further high-quality research, including future well-designed RCTs, is needed to establish optimal training dosages, determine the most effective order and combination of rehabilitation approaches, evaluate the long-term sustainability of functional gains, and develop standardised clinical practice guidelines for visual field loss after ABI.

Future research should prioritise large multicentre randomised controlled trials using standardised intervention protocols and outcome measures to facilitate comparisons across studies and strengthen the evidence base. Longer follow-up periods are also needed to determine the durability of treatment effects and their long-term impact on functional performance and quality of life. In addition, emerging technologies, including virtual reality, artificial intelligence-assisted rehabilitation and eye-tracking systems, offer promising opportunities to deliver more personalised, adaptive, and accessible rehabilitation programmes. Further research is required to establish their clinical effectiveness and define their role within routine rehabilitation pathways.

## 5. Conclusions

This systematic review suggests that visual rehabilitation interventions can improve functional outcomes in patients with visual field loss following ABI, although treatment effectiveness appears to differ across intervention types. Compensatory approaches provided the most consistent evidence of benefit, particularly in visual exploration, mobility, reading performance, and activities of daily living. In contrast, restorative interventions demonstrated more heterogeneous findings, whereas evidence remains limited for substitutive approaches and for neuromodulation used as an adjunct to restorative interventions. Importantly, functional improvements were more consistently observed than objective visual field expansion across the included studies, suggesting that successful rehabilitation may depend not only on sensory recovery but also on the development of adaptive visual behaviours. These findings underscore the need for an integrated and individualised rehabilitation approach that combines precise assessment, evidence-based intervention selection, and long-term follow-up. Future studies should also evaluate the extent to which sensory improvements translate into meaningful functional gains.

## Figures and Tables

**Table 2:** PEDro assessment of RCTs.

Author/Year	1	2	3	4	5	6	7	8	9	10	11	Total
**de Haan et al., 2015** [17]	Y	Y	N	Y	N	N	Y	Y	N	Y	Y	6
**Elshout et al., 2016** [30]	Y	Y	Y	Y	N	N	N	Y	N	Y	Y	5
**Rowe et al., 2017** [34]	Y	Y	Y	Y	Y	N	N	Y	Y	Y	Y	8
**Crotty et al., 2018** [12]	Y	Y	Y	Y	N	N	Y	N	Y	Y	Y	7
**Cavanaugh et al., 2021** [41]	Y	Y	N	Y	Y	N	Y	Y	N	Y	Y	7
**Namgung et al., 2024** [24]	Y	Y	Y	Y	Y	N	Y	Y	Y	Y	Y	9
**Rowe et al., 2025** [47]	Y	Y	Y	Y	Y	N	Y	N	N	Y	Y	7
**Namgung et al., 2025** [25]	Y	Y	Y	Y	Y	Y	Y	Y	Y	Y	Y	10
**Diana et al., 2025** [45]	Y	Y	Y	Y	Y	Y	Y	Y	Y	Y	Y	10

Yes (Y); No (N). 1. Eligibility criteria (not included in the total PEDro score); 2. Random allocation; 3. Allocation concealment; 4. Baseline comparability; 5. Blinding of subjects; 6. Blinding of therapists; 7. Blinding of assessors; 8. >85% follow-up; 9. Intention to treat analysis; 10. Between-group comparisons; 11. Point estimates and measures of variability.

**Table 3:** Risk of bias assessment of quasi-experimental studies.

Author (Year)	Q1	Q2	Q3	Q4	Q5	Q6	Q7	Q8	RoB	Q9	Sel/All	Conf	Co-Care	Measur	Attri
**Grasso et al., 2016** [31]	Y	N	Y	Y	YY	Y	Y	U	**6**	Y	H	L	L	L	U
**Sahraie et al., 2016 Exp1** [32]	Y	Y	Y	U	YY	Y	Y	U	**6**	Y	M	L	L	L	U
**Sahraie et al., 2016 Exp2** [32]	Y	N	Y	U	NN	Y	U	U	**3**	Y	H	L	L	M	U
**Bergsma et al., 2017** [33]	Y	N	Y	Y	YY	Y	Y	U	**6**	Y	M	L	L	L	L-M
**Cavanaugh& Huxlin, 2017** [19]	Y	Y	Y	Y	PN	Y	Y	Y	**7**	Y	M	L	L	L-M	L
**Turton et al., 2018** [36]	Y	N	Y	U	NN	Y	Y	Y	**5**	U	H	L	L	M	L
**Alber et al., 2017** [35]	Y	Y	U	U	NN	Y	Y	Y	**5**	Y	M	M	M	M	L
**Smaakjær et al., 2018** [37]	Y	N	Y	U	NN	Y	U	Y	**4**	Y	H	L	L-M	M	L
**El Nahas et al., 2019** [38]	Y	N	Y	U	NN	Y	Y	N	**4**	U	H	L	L	M	H
**Sahraie et al., 2020** [39]	Y	N	Y	U	NN	Y	Y	Y	**5**	Y	M	L	L	L-M	L
**Szalados et al., 2021** [40]	Y	N	Y	U	NY	Y	Y	N	**5**	Y	H	L	L	L	M-H
**Mena-García et al., 2021** [42]	Y	Y	Y	Y	NN	Y	Y	Y	**7**	Y	L	L-M	L	L-M	L
**Ajina et al., 2021** [20]	Y	Y	Y	U	NN	Y	Y	Y	**6**	Y	M	L	M	L-M	L
**Leitner et al., 2023** [23]	Y	N	Y	U	NY	Y	Y	Y	**6**	Y	H	M	M	L-M	L
**Gupta et al., 2024** [44]	Y	Y	Y	Y	YY	Y	Y	N	**7**	U	H	L	L	L	H
**Varalta et al., 2025** [46]	Y	Y	N	U	NN	Y	Y	U	**4**	Y	H	H	M	L-M	U
**Toh et al., 2026** [48]	Y	N	Y	U	NY	Y	Y	Y	**6**	Y	H	L	L	L-M	L
**Otero-Curráset al., 2026** [49]	Y	Y	N	U	NN	Y	Y	Y	**5**	Y	H	M	L	L-M	L
**Jefferson et al., 2026** [50]	Y	N	Y	U	NN	Y	U	Y	**4**	Y	H	L	L	M	L

Domains of internal validity: (Q1) Temporal precedence; (Q2) Selection and Allocation; (Q3) Cofounding Factors; (Q4) Administration of intervention/exposure (similarity of treatment/care); (Q5) Multiple outcomes measurements pre and post (e.g., YY = repeated pre/post; PN = repeated pre in one group only); (Q6) Outcomes measured in the same way; (Q7) Outcomes measured reliably; (Q8) Participants Retention; (Q9) Statistical Conclusion Validity. Y = Yes; N = No; U = Unclear; P = Partially. L = Low; M = Moderate; H = High. Sel/All = Selection and Allocation; Conf = Confounding; Co-Care = Concurrent Care; Measur = Measurement (assessment, detection and measurement of the outcome from Q5–6–7); Attri = Attrition. Methodological appraisal summaries: Appendix A, Table A2.

**Table 4:** Methodological quality assessment of case series (JBI checklist).

Study	Score	Q1	Q2	Q3	Q4	Q5	Q6	Q7	Q8	Q9	Q10
**Metzler et al., 2021** [43]	8/10	Y	U	Y	Y	U	Y	Y	Y	Y	Y

Yes (Y); No (N); Unclear (U). Q1. Clear inclusion criteria; Q2. Standard and reliable measurement of the condition; Q3. Valid methods for condition identification; Q4. Consecutive inclusion; Q5. Complete inclusion; Q6. Clear reporting of participant demographics; Q7. Clear reporting of clinical information; Q8. Clear reporting of outcomes/follow-up; Q9. Clear reporting of presenting site(s)/clinic(s); Q10. Appropriate statistical analysis. Methodological appraisal summaries: Appendix A, Table A3.

## Data Availability

The data presented in this study are available on request from the corresponding author.

## Abbreviations

The following abbreviations are used in this manuscript:

ABI	Acquired brain injury
rTMS	Repetitive transcranial magnetic stimulation
tDCS	Transcranial direct current stimulation

## Appendix A

**Table A1:** Summary of main outcome measures for included studies.

Author (Year)		Intervention	VF Recov	Visual Skills	Reading	Func Perf	QoL	Comparator
De Haan et al., 2015 [17]		Comp	—	N	N	Y+	Y+	✔
Elshout et al., 2016 [30]		Comp/Rest	Y+	—	Y+	—	—	✔
Grasso et al., 2016 [31]		Comp	—	Y	Y	Y	—	
Sahraie et al., 2016 [32]	Exp 1	Comp	—	Y+	—	Y+	—	✔
	Exp 2	Comp	—	Y	—	Y	—	
Bergsma et al., 2017 [33]		Rest	Y+	—	Y+	Y+	—	✔
Cavanaugh & Huxlin, 2017 [19]		Rest	Y+	Y+	—	—	—	✔
Rowe et al., 2017 [34]		Comp/Subs	N	—	N	N	Y+	✔
Alber et al.; 2017 [35]		Comp/Rest	Y+	—	—	—	—	✔
Turton et al., 2018 [36]		Comp	—	Y	—	Y	Y	
Crotty et al., 2018 [12]		Comp	—	N	N	N	Y+	✔
Smaakjær et al., 2018 [37]		Comp	—	Y	Y	Y	Y	
El Nahas et al., 2019 [38]		Rest	Y	—	—	—	Y	
Sahraie et al., 2020 [39]		Comp	—	Y	—	Y	—	
Szalados et al., 2021 [40]		Comp	N	Y	—	Y	—	
Cavanaugh et al., 2021 [41]		Rest	L	Y+	—	—	N	✔
Mena-García et al., 2021 [42]		Comp	L	Y+	Y+	Y+	Y+	✔
Ajina et al., 2021 [20]		Comp/Rest	L	Y+	—	—	—	✔
Metzler et al., 2021 [43]		Comp	—	Y	—	Y	—	
Leitner et al., 2023 [23]		Rest	N	L	—	—	—	
Gupta et al., 2024 [44]		Comp/Subs	—	Y+	Y=	—	—	✔
Namgung et al., 2024 [24]		Rest	Y=	Y+	—	—	—	
Diana et al., 2025 [45]		Comp	—	Y+	—	Y=	—	✔
Namgung et al., 2025 [25]		Rest	Y+	—	—	—	—	✔
Varalta et al., 2025 [46]		Comp	—	Y=	—	Y=	—	✔
Rowe et al., 2025 [47]		Comp	N	Y=	—	Y=	Y=	✔
Toh et al., 2026 [48]		Comp	—	Y	—	Y	—	
Otero-Currás et al., 2026 [49]		Comp	N	N	—	L	—	✔
Jefferson et al., 2026 [50]		Comp	—	L	—	Y	Y	

**Abbreviations.** Comp: compensatory intervention; Rest: restorative intervention; Subs: substitutive intervention; VF Recov: visual field recovery; Func Perf: functional performance (ADL, PROMs); QoL: quality of life; ✔, comparator present. **Outcome coding.** Y+: significant improvement favouring the intervention; Y=: significant improvement in both groups, with no between-group difference; Y: significant improvement in studies without a comparator; L: limited or localised improvement; N: no significant improvement; —: outcome not assessed.

**Table A2:** Detailed risk-of-bias and quality assessment for non-randomised studies.

Author/Year		RoB Q1–8	Appraisal Comments & Risk Justification	Main Sources of Bias	Statistical Conclusion Validity (Q9) *
**Grasso et al., 2016** [31]		RoB 6	Single-group pre–post design without an independent control group; small sample size (*n* = 10). Multiple pre- and post-intervention assessments supported the evaluation of baseline stability and treatment effects. Although follow-up was complete, some secondary outcome data were excluded from the statistical analyses without a formal assessment of their potential impact.	Selection/Allocation: High; Attrition: Unclear	Yes. Appropriate statistical analyses, including repeated-measures ANOVA, Greenhouse–Geisser corrections, and effect size reporting.
**Sahraie et al., 2016** [32]	**Exp1**	RoB 6	Controlled study used a historical (non-concurrent) matched control group from a prior database, raising concerns about selection. Multiple pre- and post-intervention assessments supported evaluation of baseline stability and treatment effects. A brief intervention period (mean 13 sessions; 2 weeks) limits the influence of concurrent care. Incomplete reporting of participant flow limits assessment of potential attrition.	Selection/Allocation: Moderate; Attrition: Unclear.	Yes. Appropriate statistical analyses were performed, including two-way repeated-measures ANOVA (with Greenhouse–Geisser corrections) and paired *t*-tests with Cohen’s *d* reporting.
	**Exp2**	RoB 3	Single-group pre–post design without a control group. A brief intervention period (mean 14 sessions) limits the influence of concurrent care. The study relied on a single pre- and post-intervention assessment. Outcome measurement was limited by the low sensitivity of the computerised cancellation task and the lack of oversight during home-based testing (*n* = 15). Incomplete reporting of participant flow limits assessment of attrition.	Selection/Allocation: High; Measurement: Moderate; Attrition: Unclear	Yes. Appropriate use of two-way repeated-measures ANOVA and paired *t*-tests with Bonferroni correction for multiple comparisons.
**Bergsma et al., 2017** [33]		RoB 6	The lack of an independent control group was partially mitigated by a within-subject crossover design (using the untrained visual field as an internal control), repeated pre- and post-intervention assessments, and separate analyses of subacute and chronic participants to account for spontaneous recovery. Outcome measurement reliability was supported by Goldmann perimetry with continuous fixation monitoring. Protocol-related exclusions and incomplete secondary outcome data (Goal Attainment Scaling (GAS)) resulted in a Low-Moderate attrition risk.	Selection/Allocation: Moderate. Attrition: Low-Moderate	Yes. Appropriate and robust statistical analyses, including repeated-measures factorial ANOVA with effect size reporting (partial η^2^), Wilcoxon signed-rank tests, linear regression analyses, and transparent handling of missing data through analysis-specific sample sizes and appropriate degrees of freedom.
**Cavanaugh & Huxlin, 2017** [19]		RoB 7	Small and unbalanced control group (*n* = 5) without an active control condition. Repeated baseline measurements were available for the control group to ensure pre-training defect stability. However, multiple post-intervention assessments were not performed.	Selection/Allocation: Moderate	Yes. Comprehensive and robust analyses, including paired and independent *t*-test, one-way ANOVA, 95% confidence intervals, and post hoc power analyses. Missing data and outlier analyses were reported transparently, with appropriate adjustment for sample size and degrees of freedom.
**Alber et al., 2017** [35]		RoB 5	Pilot study using retrospectively selected matched controls, which may have differed in lesion topography despite matching for age, lesion time, and baseline visual field sensitivity. Despite the brief intervention period (10 sessions over 28 days), the influence of concurrent rehabilitation and spontaneous recovery during the subacute post-stroke phase cannot be excluded. The study relied on a single pre- and post-intervention assessment. Perimetric fixation control relied on central detection tasks, which have well-recognised technical limitations.	Selection/Allocation: Moderate; Confounding: Moderate; Concurrent Care: Moderate; Measurement: Moderate.	Yes. Appropriate and comprehensive statistical analyses were performed, including non-parametric statistical analyses (Wilcoxon signed-rank and rank-sum tests) for the small sample size. The analysis was strengthened by Pearson correlation analyses to identify baseline predictors of recovery and by detailed evaluation of absolute and relative visual field improvements.
**Turton et al., 2018** [36]		RoB 5	Single-group pre–post design without a control group and with a single pre- and post-intervention assessment. A brief intervention period (9 sessions over 3 weeks) limits the potential influence of concurrent care. The static nature of the room-search task may not fully reflect visual search behaviour in real-world activities.	Selection/Allocation: High; Measurement: Moderate.	Unclear. Appropriate descriptive statistics were used (medians, IQR, and 95% CI) for the study’s pilot and feasibility objectives. However, the small, heterogeneous sample (*n* = 9) resulted in limited statistical power, precluding robust conclusions regarding intervention effectiveness.
**Smaakjær et al. (2018)** [37]		RoB 4	Single-group open-label design without a control group and with a single pre- and post-intervention assessment. The primary outcome (COPM) was assessed by the treating therapist, increasing the potential for expectancy and detection bias. The inclusion of objective performance-based secondary outcomes partially mitigated this risk, while the chronic cohort (median 9 months post-stroke) reduced the likelihood of confounding due to spontaneous recovery.	Selection/Allocation: High; Concurrent Care: Low-Moderate; Measurement: Moderate.	Yes. Appropriate statistical analyses were performed, including a priori sample size calculation and Wilcoxon tests for paired non-parametric data. Missing data were handled transparently, and analyses were performed by an independent statistician.
**El Nahas et al., 2019** [38]		RoB 4	Open-label pilot study design without an independent control group and with a single pre- and post-intervention assessment. A brief intervention period (16 sessions over 1 month) limits the potential influence of concurrent care. Substantial missing data for the primary outcome (8/12 participants completed the final assessment), without formal analysis of the impact of missing data.	Selection/Allocation: High; Measurement: Moderate; Attrition: High.	Unclear. Appropriate statistical analyses were reported, but the final analysis was based on only eight participants. No a priori sample size or power calculation, assessment of statistical assumptions, effect sizes, or adjustment for potential confounding were reported.
**Sahraie et al., 2020** [39]		RoB 5	Large prospective single-group study without an independent control group and with a single pre- and post-intervention assessment. Home-based delivery and self-reported diagnosis limited independent verification of participant characteristics. A brief intervention period (median 23 days) in a predominantly chronic cohort (mean 1.84 years post-stroke) reduced the potential influence of concurrent care. Primary outcomes were recorded automatically by the NeuroEyeCoach™ software with objective computer logging and rigorous data handling. Incomplete self-reported disability data affected only a secondary outcome.	Selection/Allocation: Moderate; Measurement: Low-Moderate.	Yes. Appropriate statistical analyses were performed, including *t*-tests, ANOVA, and regression analyses aligned with the study objectives. Effect sizes were reported, potential confounding variables were considered, and median reaction times were analysed to reduce the influence of outliers. The large sample (*N* = 296) supported robust statistical inference, and the project was pre-registered on OSF.
**Szalados et al., 2021** [40]		RoB 5	Large prospective web-based cohort study without an independent control group. The authors acknowledge that the sample may not represent typical clinical populations. Repeated post-intervention assessments supported objective outcome measurement. A brief intervention period (median 20 days) in a predominantly chronic cohort reduced the potential influence of concurrent care. Only participants who completed all 1200 therapy trials were analysed, while intermediate data from non-completers were not, introducing potential completer (survivor) bias.	Selection/Allocation: High; Attrition: Moderate–High (completer bias)	Yes. Appropriate statistical analyses were performed, including repeated-measures ANOVA with within-subject and between-subject factors, Greenhouse–Geisser correction when required, post hoc group analyses, and both standardised and non-standardised effect size reporting.
**Mena-García et al., 2021** [42]		RoB 7	Non-randomised matched-group design with groups matched for age, brain damage duration, and visual field defect type, despite a significant baseline difference in visual acuity. The intervention group underwent an additional mid-training assessment, allowing evaluation of the temporal trajectory of treatment effects. Although this assessment was not performed in the control group, sensitivity analyses demonstrated no significant learning effect. Outcome assessment was not blinded despite the use of objective and standardised measurement procedures.	Confounding: Low-Moderate; Measurement: Low-Moderate.	Yes. Appropriate statistical analyses were performed according to the data type, including Student’s *t*-tests, Mann–Whitney U, χ^2^/Fisher tests, repeated-measures ANOVA, Friedman and Wilcoxon tests. Effect sizes (Cohen’s d) and 95% CIs were reported, and a sensitivity analysis assessed the potential influence of the additional mid-intervention assessment.
**Ajina et al., 2021** [20]		RoB 6	Small quasi-experimental study with a very small control group (*n* = 4). Groups were comparable at baseline for age and time since lesion. The study relied on a single pre- and post-intervention assessment. Concurrent rehabilitation or visual interventions were not monitored. Outcome measurement relied on highly reliable objective measures, including standardised psychophysical testing, fMRI, and eye-tracking. However, fMRI outcomes were acquired only in the intervention group, preventing comparison with controls and limiting the exclusion of placebo-related effects, as acknowledged by the authors.	Selection/Allocation: Moderate Confounding: Moderate; Concurrent Care: Moderate Measurement: Low-Moderate	Yes. Appropriate and rigorous statistical analyses were performed, including repeated-measures two-way ANOVA to assess training effects and Spearman’s rank correlation analyses to examine associations between behavioural improvements and neural activity. Neuroimaging analyses incorporated robust methods with appropriate multiple-comparisons correction, strengthening the validity of the findings.
**Leitner et al., 2023** [23]		RoB 6	Clinical pre-post intervention study without an independent control group. Multiple post-intervention assessments over the 5-month intervention allowed evaluation of the temporal trajectory of treatment effects. Outcome measurement relied on a validated eye-tracking-based perimetric method that objectively controlled fixation in real time, minimising measurement bias. Concurrent rehabilitation or visual interventions were neither reported nor monitored.	Selection/Allocation: High; Confounding: Moderate; Concurrent Care: Moderate.	Yes. Appropriate and robust statistical analyses were performed, including paired *t*-tests and Kolmogorov–Smirnov tests for normality. Effect sizes (*r*) were reported, and the analysis was strengthened by an eccentricity-weighted perimetric approach and the comparison of objective outcomes with validated self-reported visual function (VAS).
**Gupta et al., 2024** [44]		RoB 7	Quasi-randomised clinical trial. A substantial proportion of participants (20/50, 40%) did not complete the initial prism adaptation phase and were therefore not allocated to the intervention groups, increasing the risk of selection and attrition biases. Multiple pre- and post-intervention assessments supported evaluation of baseline stability and treatment effects. Outcome assessment was performed by masked independent examiners.	Selection/Allocation: High; Measurement: Moderate; Attrition: High.	Unclear. Appropriate statistical analyses were performed in accordance with the study design, including independent *t*-tests, repeated-measures ANOVA with Bonferroni post hoc comparisons, and Mann–Whitney U tests. However, no sample size calculation, effect sizes, or adjustment for potential confounding factors were reported.
**Varalta et al., 2025** [46]		RoB 4	Quasi-experimental study comparing supervised and home-based delivery of the same rehabilitation programme. Marked baseline differences between groups increased the risk of selection bias. Participants in the supervised group attended daily neurorehabilitation; concurrent interventions in the home-based group were neither reported nor controlled for. The study relied on a single pre- and post-intervention assessment. Outcome assessment relied on objective, standardised and automated measures applied consistently across groups. Only participants who completed the rehabilitation programme were included in the analysis, but participant losses during follow-up were not reported.	Selection/Allocation: High; Confounding: High; Concurrent Care: Moderate; Measurement: Low-Moderate; Attrition: Unclear.	Yes. Appropriate statistical analyses were performed, including repeated-measures ANOVA to evaluate training and group × training effects, paired *t*-tests for within-group comparisons, and Bonferroni correction for post hoc analyses. Effect sizes (Cohen’s d and partial η^2^) were reported, strengthening the interpretation of the findings.
**Toh et al., 2026** [48]		RoB 6	Single-arm clinical trial without an independent control group. A brief intervention period (3 sessions) limits the influence of concurrent care. Multiple post-intervention assessments, but only a single baseline measurement, allowed evaluation of the temporal trajectory of treatment effects. Outcome assessment relied on objective, standardised computer-based measures.	Selection/Allocation: High; Measurement: Low-Moderate.	Yes. Robust statistical analyses, including an a priori power calculation (*N* = 16), GLMM/LMM for nested repeated-measures data, and 95% CIs. Model assumptions were satisfied through log transformations, and stable seeing-side performance supported the absence of practice effects. Hypotheses were pre-specified, and the trial was prospectively registered.
**Otero-Currás et al.; 2026** [49]		RoB 5	Controlled parallel-group study comparing dichoptic visual training with a no-treatment control group. Inconsistencies in the reporting of group allocation and baseline differences between groups increased the risk of selection and confounding biases. A brief intervention period (6 sessions) limits the influence of concurrent care. The study relied on a single pre- and post-intervention assessment. Outcome assessment incorporated objective, standardised eye-tracking-based measures applied consistently across groups, and follow-up was complete.	Selection/Allocation: High; Confounding: Moderate; Measurement: Low-Moderate.	Yes. Appropriate statistical analyses were performed, including normality testing, non-parametric comparisons (Mann–Whitney U and Wilcoxon tests), and an exploratory mixed-design ANOVA to examine group-by-time interactions. Baseline differences in stereopsis and distance deviation were appropriately excluded from the change analysis to minimise potential confounding.
**Jefferson et al.; 2026** [50]		RoB 5	Single-arm clinical trial without an independent control group. A brief intervention period (5 sessions over 2–3 weeks) limits the potential influence of concurrent care. The study was limited to a single pre- and post-intervention assessment. Outcome assessment combined objective, standardised clinical tests with a computer-based platform. However, its reliability in this clinical population remains unclear, as the Senaptec system has not been psychometrically validated in stroke survivors.	Selection/Allocation: High; Confounding: Moderate; Measurement: Moderate.	Yes. Appropriate statistical analyses were performed for the study objectives, including paired *t*-tests, repeated-measures ANOVA with Greenhouse–Geisser correction where appropriate, and effect size reporting (Cohen’s *d*).

* Q9 refers to the JBI item assessing statistical conclusion validity and was considered separately from the overall risk-of-bias assessment. Unless otherwise stated, studies did not report a sample size calculation or a formal a priori statistical power analysis. A rating of “Yes” indicates that statistical analyses were appropriate for the study design and available data.

**Table A3:** Detailed risk-of-bias and quality assessment for case series.

	Score	Appraisal Comments & Risk Justification	Main Sources Bias
**Metzler et al., 2021** [43]	8/10	Valuable ecological validity reflecting routine outpatient practice; however, results should be interpreted with caution due to potential referral bias, substantial missing data (attrition bias) in functional outcomes, the retrospective design, and the lack of an independent control group. Outcome measures were reliable but were not administered in a standard way for all participants.	Selection/Allocation: Unclear (potential referral bias); Concurrent Care: High; Measurement: Moderate (High for PROMs); Attrition: Variable (High for PROMs).
